# Supplementary material for: Highly Sensitive Detection of Benzoyl Peroxide Based on Organoboron Fluorescent Conjugated Polymers
Source: Polymers (Basel). 2019 Oct 11;11(10):1655. doi: 10.3390/polym11101655 (PMC6835668; doi:10.3390/polym11101655)
Supplement: Supplementary file 1 [file polymers-11-01655-s001.pdf]

polymers

MDPI

Supplementary Materials

Highly Sensitive Detection of Benzoyl Peroxide Based on Organoboron Fluorescent Conjugated Polymers

Mingyuan Yin [1], Caiyun Zhang [1], Jing Li [1], Haijie Li [1], Qiliang Deng [1,*] and Shuo Wang [1,2,*]

[1] State Key Laboratory of Food Nutrition and Safety, School of Food Engineering and Biotechnology, College of Chemical Engineering and Materials Science, Tianjin University of Science and Technology, Tianjin 300457, China; mingyuanyinymy@163.com (M.Y.); zcyyhdjc@163.com (C.Z.); lijingcgrs@163.com (J.L.); lihaijie198710@163.com (H.L.)

[2] Tianjin Key Laboratory of Food Science and Health, School of Medicine, Nankai University, Tianjin 300071, China

* Correspondence: yhdql@tust.edu.cn (Q.D.); s.wang@tust.edu.cn (S.W.)

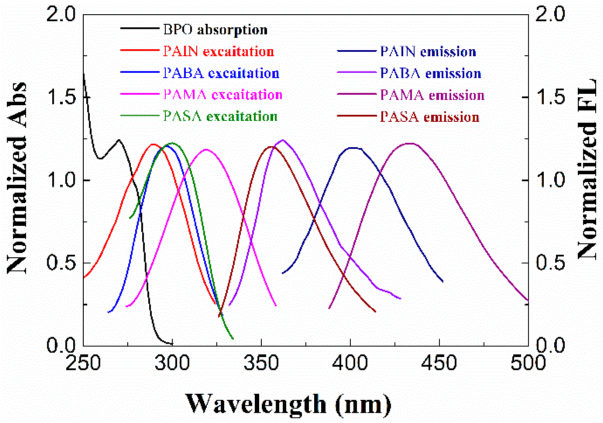

Figure 1. Normalized UV-vis absorption spectra of BPO and the fluorescence excitation and emission spectra of FCPs.

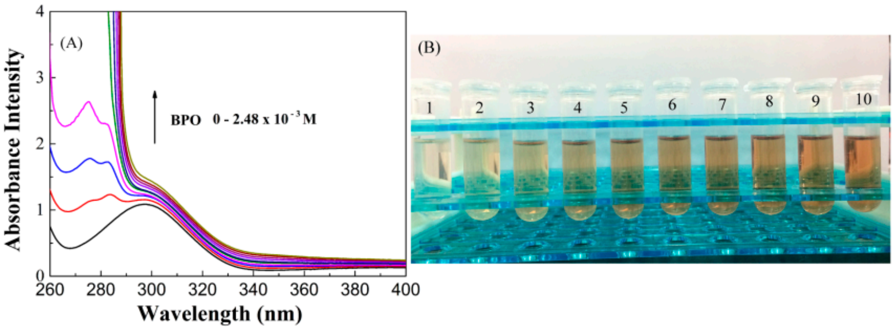

Figure 2. (A) UV-vis absorption spectra of PABA treated with BPO (0–2.48×10^{-3} M) and (B) photographs of PABA treated with BPO.

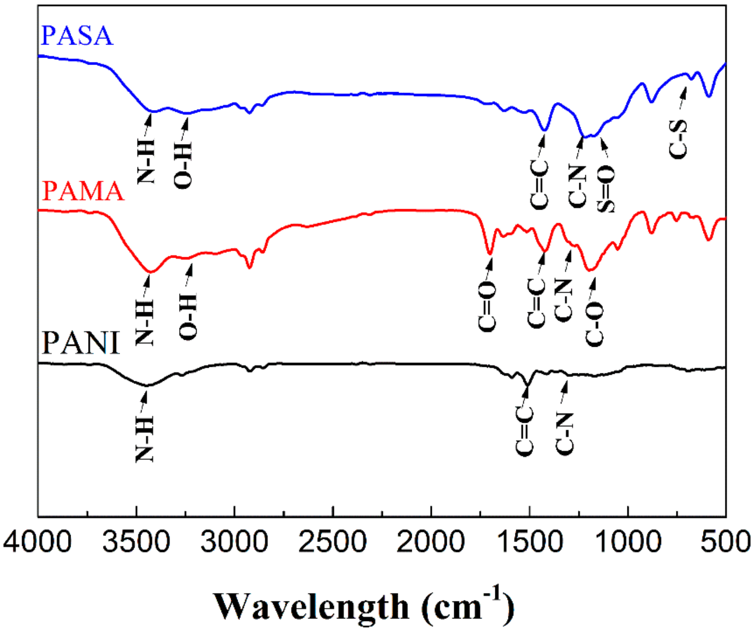

Figure 3. FT-IR spectra of PANI, PAMA, and PASA.

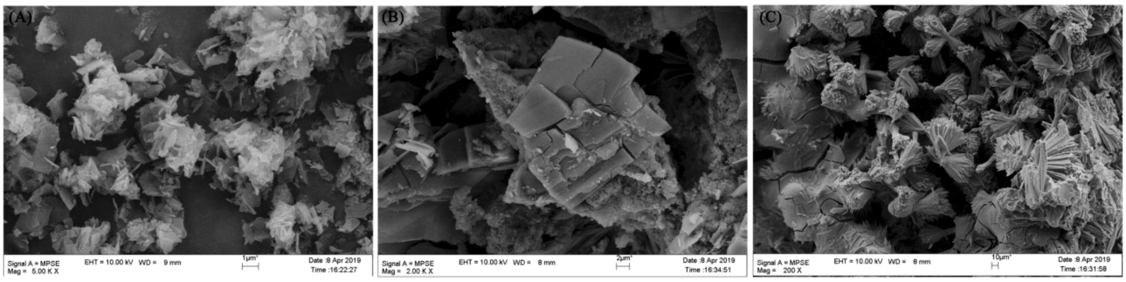

Figure 4. SEM images of (A) PANI, (B) PAMA, and (C) PASA.

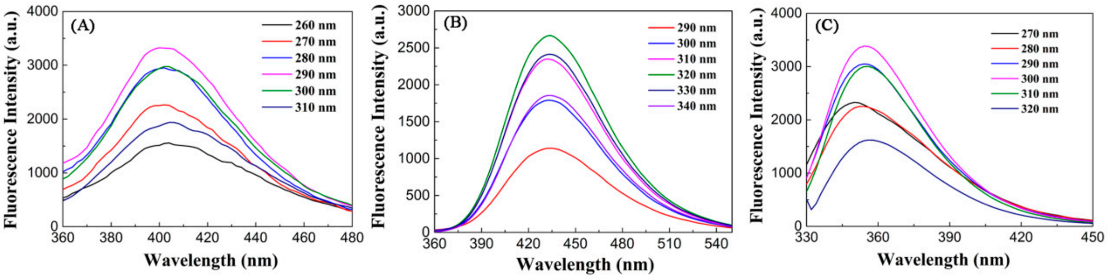

Figure 5. Fluorescence emission spectra of (A) PANI, (B) PAMA, and (C) PASA at different excitation wavelengths in ethanol.

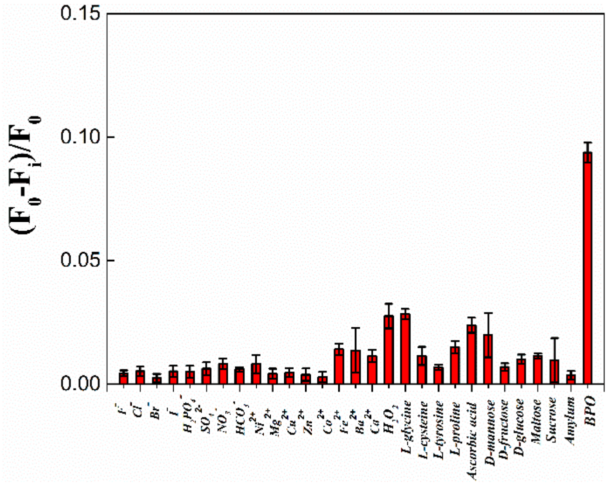

Figure 6. Comparison of the relative fluorescence intensity ($(F_0–F_i)/F_0$) of PABA (0.25 mg mL^{-1}) reacting with different substance (8.26×10^{-6} M). F_0 and F_i are the fluorescent intensity of PABA in the absence and presence of different substance, respectively.

Table 1. Elemental analyses results of the obtained FCPs.

FPCs	C %	N %	O %	S %	B %
PANI	53.13	46.87	-	-	-
PABA	55.11	8.25	22.09	-	14.55
PAMA	51.19	11.34	37.47	-	-
PASA	68.94	12.98	11.13	6.94	-

Table 2. Zeta potentials of the obtained FCPs in water (T = 25 °C, 0.25 mg mL^{-1}).

Sample	Zeta potential (mV)
PANI	1.43± 0.10
PABA	−28.4 ± 0.70
PAMA	−24.2 ± 0.91
PASA	−26.0 ± 1.00

Table 3. Comparison of molecular weight of PABA treated without/with BPO.

Sample	Treat PABA	Number Average Molecular Weight (M_n)	Weight Average Molecular Weight (M_w)	Molecular Weight Distribution (Pd)
1	Without BPO	3172	3337	1.05
2	With BPO	3338	3563	1.07

Table 4. Comparison of different methods for the determination of BPO.

Method	Linearity range (*M*)	Detection limit (*M*)	Ref.

Chromogenic substrate-based spectrophotometric	$8.26\times10^{-4} – 4.13\times10^{-3}$	1.03×10^{-4}	1
Natural reagent extracts-based spectrophotometric	$3.88\times10^{-5} – 4.13\times10^{-4}$	1.61×10^{-5}	2
Peroxidases- based amperometric	$5.00\times10^{-6} – 5.50\times10^{-5}$	2.50×10^{-6}	3
Chromatography detection	$8.26\times10^{-6} – 8.26\times10^{-4}$	1.20×10^{-6}	4
Au@Ag nanorods-based colorimetric	$0–1.00\times10^{-4}$	7.50×10^{-7}	5
Rhodamine spectroscopic probe	$8.26\times10^{-7} – 1.32\times10^{-5}$	2.48×10^{-7}	6
Ratiometric fluorescent probe	$0–1.00\times10^{-5}$	1.63×10^{-7}	7
Near-Infrared fluorescent probe	$5.00\times10^{-7}– 4.00\times10^{-6}$	4.70×10^{-8}	8
This method	$8.26\times10^{-9}– 8.26\times10^{-4}$	1.06×10^{-9}	

References

1. Ponhong, K.; Supharoek, S. A.; Siriangkhawut, W.; Grudpan, K., A rapid and sensitive spectrophotometric method for the determination of benzoyl peroxide in wheat flour samples. *J. Food Drug. Anal.* **2015,** *23* (4), 652–659.
2. Supharoek, S. A.; Ponhong, K.; Grudpan, K., A green analytical method for benzoyl peroxide determination by a sequential injection spectrophotometry using natural reagent extracts from pumpkin. *Talanta* **2017,** *171*, 236–241.
3. Kozan, J. V.; Silva, R. P.; Serrano, S. H.; Lima, A. W.; Angnes, L., Amperometric detection of benzoyl peroxide in pharmaceutical preparations using carbon paste electrodes with peroxidases naturally immobilized on coconut fibers. *Biosens. Bioelectron.* **2010,** *25* (5), 1143–8.
4. Mu, G. F.; Liu, H. T.; Gao, Y.; Luan, F., Determination of benzoyl peroxide, as benzoic acid, in wheat flour by capillary electrophoresis compared with HPLC. *J. Sci. Food Agr.* **2012,** *92* (4), 960–964.
5. Lin, T. R.; Zhang, M. Q.; Xu, F. H.; Wang, X. Y.; Xu, Z. F.; Guo, L. Q., Colorimetric detection of benzoyl peroxide based on the etching of silver nanoshells of Au@Ag nanorods. *Sens. Actuators B Chem.* **2018,** *261*, 379–384.
6. Chen, W.; Shi, W.; Li, Z.; Ma, H. M.; Liu, Y.; Zhang, J. H.; Liu, Q. J., Simple and fast fluorescence detection of benzoyl peroxide in wheat flour by N-methoxy rhodamine-6G spirolactam based on consecutive chemical reactions. *Anal. Chim. Acta.* **2011,** *708* (1-2), 84–88.
7. Hu, Q.; Li, W.; Qin, C.; Zeng, L.; Hou, J. T., Rapid and Visual Detection of Benzoyl Peroxide in Food by a Colorimetric and Ratiometric Fluorescent Probe. *J. Agr. Food Chem.* **2018,** *66* (41), 10913–10920.
8. Tian, X. W.; Li, Z.; Pang, Y. X.; Li, D. Y.; Yang, X. B., Benzoyl Peroxide Detection in Real Samples and Zebrafish Imaging by a Designed Near-Infrared Fluorescent Probe. *J. Agr. Food Chem.* **2017,** *65* (43), 9553–9558.

© 2019 by the authors. Licensee MDPI, Basel, Switzerland. This article is an open access article distributed under the terms and conditions of the Creative Commons Attribution (CC BY) license (http://creativecommons.org/licenses/by/4.0/).
